# Human amniotic membrane for the treatment of large and refractory macular holes: a retrospective, multicentric, interventional study

**DOI:** 10.1186/s40942-021-00308-6

**Published:** 2021-05-08

**Authors:** Magno A. Ferreira, André Maia, André J. Machado, Raquel E. A. Ferreira, Luiz Felipe Hagemann, Pedro Hélio E. Ribeiro Júnior, Flávio A. Rezende

**Affiliations:** 1 Universidade Federal de Uberlândia UFU - Federal University of Uberlândia, Uberlândia, Brazil; 2 Uberlândia Eye Hospital (HOLHOS), Av. Pará, 1720-Umuarama, Uberlândia, MG 38405-320 Brazil; 3 Departamento de Retina e Vítreo - Retina and Vitreous Department of the Universidade Federal de São Paulo (UNIFESP)-(Federal University of São Paulo), São Paulo, Brazil; 4 Retina Clinic, São Paulo, Brazil; 5 Universidade Federal Do Ceará (UFC) - Federal University of Ceará and President of CLDO, Fortaleza, Brazil; 6 Hospital de Olhos Blumenau - Blumenau Eye Hospital, Blumenau, Brazil; 7 Federal University of Uberlândia (UFU), Uberlândia, Brazil; 8 Department of Ophthalmology, Maisonneuve-Rosemont Hospital, University of Montreal, Montreal, Canada

**Keywords:** Macular-hole, Macular-hole reoperations, Large macular-hole, Human amniotic membrane, Macular surgery

## Abstract

**Background:**

The purpose of the current study is to report the anatomical and functional results of off-label human amniotic membrane graft as primary intervention to repair large to giant macular holes and in reoperations when wide internal limiting membrane peeling was unsuccessful.

**Methods:**

Retrospective chart review was carried out in five different centers to identify all cases that had undergone off-label human amniotic membrane graft for the treatment of large or failed macular holes (MH). Data collected included age, gender, other concomitant diagnosis, symptoms duration, lens status, number of previous surgeries, macular hole measurements (minimum and base linear diameters), mean post-operative follow-up (months), and pre- and post-operative best corrected visual acuity (BCVA). Main outcome measures were anatomical MH closure rates and final BCVA (in logMAR). Nonparametric Wilcoxon rank-sum test was used because the data was not normally distributed, a P values < 0.05 were considered statistically significant.

**Results:**

Nineteen eyes of 19 patients were identified and included in the study. Mean age was 66.21 ± 14.96 years and predominantly females (84%). All eyes had successfully closed MH with a single intervention with no recurrences during a mean of 9 ± 3.87 months follow-up. The median BCVA in logMAR preoperative was 1.30 ± 0.44 (0.80–2.0), approximately 20/400 on Snellen chart and the median BCVA in logMAR postoperative was 1.0 ± 0.72 (0.4–3.0) approximately 20/200 (p < 0.0001) with median of three lines of visual improvement.

**Conclusion:**

The use of human amniotic membrane graft seems to be a viable and effective alternative for the treatment of large and persistent macular holes. However, further larger prospective controlled studies are necessary to confirm our preliminary results of this new surgical technique.

## Background

Macular holes (MHs) are full-thickness defects in the outer plexiform and photoreceptor layers at the fovea, causing impaired central vision with metamorphopsia. Macular hole prevalence is 3.3 in 1000 in people older than 55 years old. Patients are usually in their sixth or seventh decade of life; approximately two-thirds of patients are female [1]. Nearly one-half of MHs are large (defined as an MH with a minimum linear diameter [MLD] of > 400 μm at the time of the diagnosis [2]. A review of MH previous therapies and pathogenesis provides a better understanding of their pathophysiology and allows the development and improvement of new interventions. Following the pathogenetic theory described by Gass [3], Kelly and Wendel reported in 1991 the successful closure of an MH in 30 of 52 (58%) patients with MHs using vitrectomy and gas [4]. Initial skepticism for the surgical treatment of MH [5] was replaced by enthusiasm as success rates surged with increasing surgeon experience and improved techniques. Eckhart et al. [6] and Park et al. [7] reported that the MH closure rate could be improved by adding internal limiting membrane (ILM) peeling delamination to the surgical procedure. Subsequently, ILM staining with 0.06% indocyanine green dye enhanced ILM identification, allowing for more efficient ILM delamination [8]. A randomized, controlled trial demonstrated higher anatomic closure and lower reoperation rates in patients who had macular ILM peeling vs those that did not [9]. Overall, anatomical closure rates of MHs have been reported to be as high as 85% to 100% [10, 11]. Macular hole diameter measurements calculated by Optical Coherence Tomography (OCT) may be used to predict anatomic surgical success with standard ILM peeling surgery, which in turn is correlated with functional improvement. The most basic, MH minimum linear diameter (MLD), classifies “large MH” as those with an MLD > 400 μm, which have poorer prognosis or surgical success than those < 400 μm, as described above. There is epidemiologic data supporting the use of MH MLD as a guide for surgical selection in patients with MHs. In the Manchester Large Macular Hole Study, the anatomical success rate of type 1 closure (closed without foveal neurosensory retinal defect) of full thickness macular holes (FTMH) with “standard” ILM peeling delamination varies from 91% (59/65) to 98% (64/65) in MHs between 400–649 μm in MLD, and 76% (49/64) in those from 650–1416 μm MLD. Based on the evaluation of this data, the authors proposed that an MH MLD of < 650 μm could be used as an optimal “inflection marker” to predict when MHs are more likely to close with standard surgery [12]. When the independent variable MH base diameter was considered separately, the failure rate was 0% among eyes with base-hole diameter less than 500 μm and 19.1% with a base-hole diameter of 1.000 μm or greater (P = 0.001). Several different techniques have been described to attempt large MH closure when posterior hyaloid detachment and ILM peeling were unsuccessful, such as repeat fluid-gas exchange [13], endotamponade with silicone oil [14], radial relaxing retinotomy on the MH margin [15], perifoveal laser photocoagulation to form chorioretinal adhesions [16], temporal scleral imbrication [17], autologous ILM flap [18], addition of autologous blood to the autologous ILM flap, autologous anterior or posterior lens capsule flap [19], episcleral posterior buckling [20], adjuvant blood components including platelet-rich plasma [21], suprachoroidal buckling and scleral shortening techniques [22], subretinal fluid application [23] or the more recently introduced autologous retinal transplant for refractory macular hole [24]. The above-mentioned procedures have gained from 60 to 90% of anatomical success and a BCVA improvement of 80% in the case of anatomical success. The human amniotic membrane (hAM) is the innermost layer of the fetal membranes; it has a stromal matrix, a thick collagen layer, and an overlying basement membrane with a single layer of epithelium [25]. The hAM is a basement membrane with antiangiogenic and anti-inflammatory properties well known in anterior segment reconstructive surgery. The use of the amniotic membrane transplantation to treat ocular surface abnormalities was first reported seven decades ago [26]. In these cases, hAM works as an optimal biological support for conjunctival cell growth. Kiilgaard demonstrate that hAM is well tolerated in the subretinal space of pigs, without evidence of inflammation [27], In vivo hAM plug transplantation was recently used for choroidal hole repair in a case of globe rupture [20]. Caporossi et al. and Rizzo et al. [28, 29] demonstrated in humans the use of hAM to repair retinal detachment associated with large macular tear, showing in these complex cases that hAM transplantation can be a valid option not only to help the retinal reattachment but also for a partial regenerative effect which, in these cases, was accompanied by visual acuity improvement. No retina or vitreous inflammation was noted in this study.

This study aims at analyzing the anatomical results of type I closure of the MH, as a primary outcome, with the off-label use of hAM in large macular holes (MH MLD greater than 400 microns) or reoperations where ILM peeling has already been done, whose surgical results are poor, both anatomical and functional with the different techniques, or had been done without closing the hole. As a secondary outcome, we will evaluate the functional outcome in cases of closed macular hole (BCVA before and after surgery).

## Methods

This was a multicentric, retrospective, interventional, consecutive case series conducted at five different eye hospitals and surgeons in Brazil and one in Canada, Uberlândia—Ferreira, M.A., Sao Paulo—Maia, A., Fortaleza—Machado, A.J., Blumenau—Hagemann, L.F. and Montreal—Rezende, F.A. The “off label” use of amniotic membrane for macular surgery was discussed with the patients and all patients sign an informed consent for the procedure. Due to the poor prognosis with the conventional techniques for these cases and few options to have the macular hole close, all patients agreed with the procedure. The study adheres to the tenets set forth in the declaration of Helsinki.

The obtaining, preparation and preservation of the amniotic membrane were carried out in accordance with the rules of the protocol of the ethics committee of each institution involved. The amniotic membrane was obtained from placentas from elective cesarean sections of patients who signed the consent form. All of these patients had negative serological tests for HIV-1, Hepatitis B (HBsAg) and syphilis (VDRL), which were re-confirmed by performing umbilical cord blood serology after delivery. After obtaining the placenta, it was washed with 0.9% saline in a sterile environment. With the aid of sterile scissors and tweezers, the amnion was separated from the chorion, spreading it over a sterile nitrocellulose filter with the epithelial face upwards. The amniotic membrane and the filter were washed with phosphate buffer solution containing 1000 U/ml penicillin, 20 mcg/ml streptomycin and 2.5 mcg/ml amphotericin B, cut into fragments of approximately 10 × 10 cm, placed in a sterile container containing glycerol and corneal preservation in a 1: 1 ratio and frozen at − 80 °C. Samples of each membrane obtained were sent for anatomopathological and microbiological study (bacterioscopy and culture) for quality control. The maximum time for using the membranes was 4 months after freezing [30].

The inclusion criteria for this study were patients that had large macular holes (MLD > 400 microns) or were reoperated, in which an extensive ILM peeling has already been done. Any etiology for the macula hole was accepted (eg: idiopatic, high myopia, etc.). Thus, 19 patients were selected in 5 different centers, simulating something that we normally have in real life and leads to difficulties in closing the macular hole either because of its size or because it has already been tried previously and failed or both. These patients underwent a standard 3-port, 23 or 25-gauge transconjunctival pars plana vitrectomy (Alcon Laboratories, Fort Worth, TX) and a 4-port was used to allow bimanual surgery, under retrobulbar or peribulbar anesthesia and hAM plug transplantation in the epi or subretinal space in the macular hole. The technique for introducing the hAM into the vitreous cavity, placing it on or inside the macular hole was not standard. Preoperatively, an ophthalmic history and a complete ophthalmic examination including refraction with assessment of best-corrected visual acuity (BCVA in Snellen, and transformed in logarithm of the minimal angle of resolution [logMAR]), Goldmann applanation tonometry, standard fundus dilated ophthalmic examination, spectral domain OCT (AngioVue Optovue, Fremont, CA or Spectralis HRA-OCT, Heidelberg, Germany) analysis of the macular hole with an accurate measurement of the minimum linear diameter (MH MLD) and basal diameter (MH BD). A complete vitrectomy was performed with vitreous base shaving (Constellation; Alcon Surgical, Dallas Fort Worth, TX). A chandelier endoilluminator was inserted to facilitate bimanual maneuvers. A hAM plug was taken from the hAM patch, and the final dimensions were adjusted with 2–3 mm dermatological trephine, depending on the size of the macular hole (MLD and MH BD). The hAM plug was flipped and rolled inside the vitreoretinal forceps and inserted through the trocar into the vitreous chamber. Within the vitreous cavity, hAM was manipulated under BSS with the use of valve trocars and closed infusion to prevent movement of the membrane flap in the vitreous cavity upon release of the forceps. After positioning the stromal side of the flap in epi or subretinal space, fluid–air exchange and 14% perfluoropropane gas (C3F8) or 20% sulfur hexafluoride (SF6) was used as an endotamponade. The guarantee that the flap remained intact is only obtained after performing OCT which was performed in the postoperative follow-up. The closure of the MH was considered when type 1 closure (closed without foveal neurosensory retinal defect) was obtained, which was evaluated with OCT.

Analyses were performed using GraphPad (Prism., San Diego, CA). Recorded Early Treatment of Diabetic Retinopathy Study and Snellen VA were converted to logarithm of the minimum angle of resolution (logMAR) VA. Visual improvement was defined as an increase of at least 0.3 logMAR units, and decline was defined as a decrease of at least 0.3 logMAR units (equivalent to 15 Early Treatment of Diabetic Retinopathy Study letters change). Descriptive statistics were computed. Nonparametric Wilcoxon rank-sum test was used because the data was not normally distributed. P values < 0.05 were considered statistically significant. All patients underwent examination at postoperative day 1, week 1, month 1, month 3, and month 6. Interim visits were as needed.

## Results

**Fig. 1:**
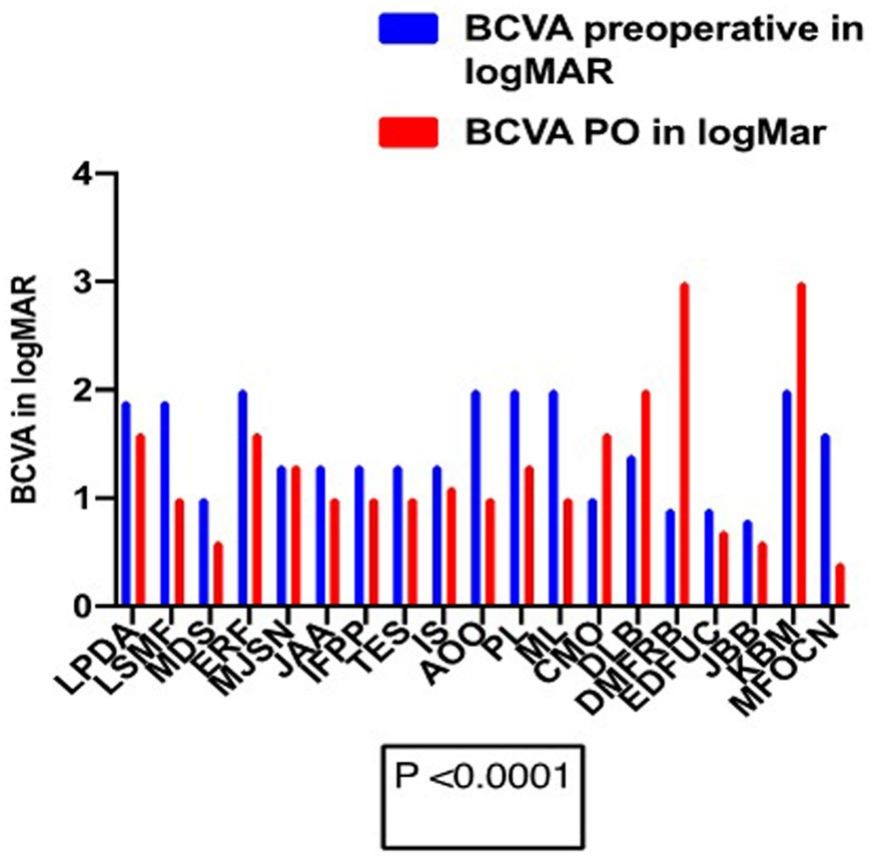
Preoperative and postoperative BCVA comparison

**Fig. 2:**
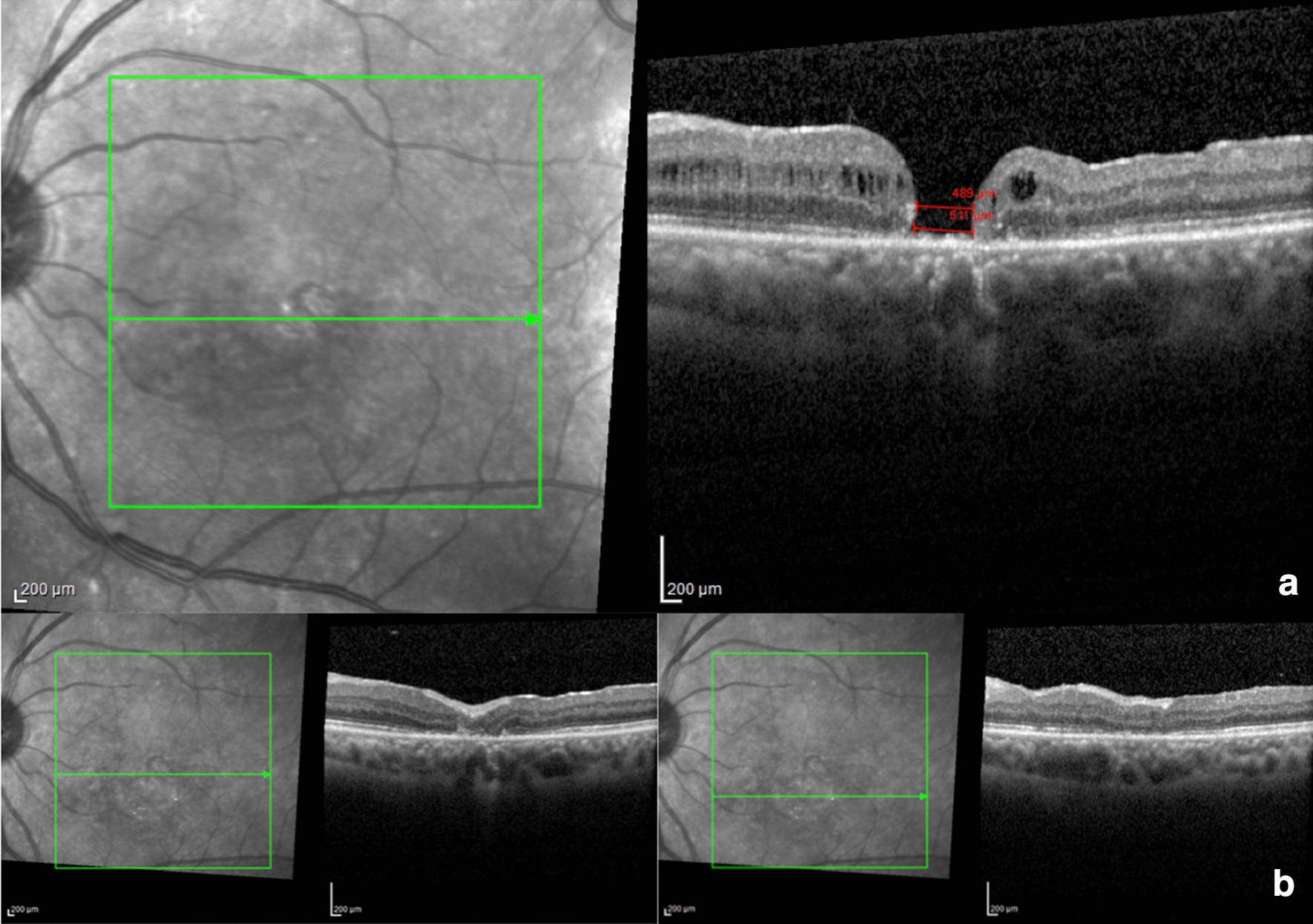
Preoperative and postoperative aspect of the OCT-SD of a patient submitted to hAM graft, over the macular hole, for the second reoperation of the macular hole. **a** Patient submitted to surgery to remove the PFC, evolute with macular hole, during the surgery, and was added to the initial procedure ILM peeling and C3F8, and in the follow-up, the macular hole was open with MLD of 489 micron, MH BD of 511 micron and BCVA of 1.9 logMAR or 20/1600. **b** One-month postoperative OCT in two cuts showing the closed macular hole with a thin layer of hAM in the internal retina and mild disruption of the ellipsoid zone and external limiting membrane in the central area with BCVA of 0.4 logMAR or 20/50

Nineteen patients were operated on from December 2018 to March 2020 in five different centers. Ferreira, M.A., had performed surgeries in six patients in Uberlandia, MG, Brazil, Maia, A., in six patients in São Paulo Brazil, Machado, A.J., in three patients in Fortaleza, CE, Brazil, Hagemann, L.F., in two patients in Blumenau, SC, and Rezende, F.A., two patients in Montreal, QC, Canada. All the findings are presented in Table 1. Sixteen patients (84.2%) were female and 3 (15.8%) male. The average age was 66.21 ± 14.96 years old (range, 23–85). Six patients were operated for the first time (32%), eight for the second time (42%) four for the third time (21%) and one for the sixth time (5%). Twelve patients were diagnosed for large macular hole (LMH) (63.20%), one patient (5.30%) developed large macular hole after subretinal injection of TPA for subretinal hemorrhage displacement, secondary to polypoidal choroidal vasculopathy (PCV) and the surgery for macular hole with ILM peeling and perfluoropropane gas (C3F8) as tamponade was added to the procedure initially proposed, but the patient, in the follow-up, remained with the macular hole open. One patient had LMH and Alport syndrome (5.30%), three patients (15.80%) LMH and high myopia and two patients (10.53%) were reoperated, but the macular hole was not classified as large macular hole. The average macular hole base diameter (MH BD) was 1301 ± 742 microns (384–2908) and macular hole minimum linear diameter (MH MLD) average was 856 ± 459 microns (301–2195). The average time of diagnosis was 27.00 ± 24.98 months (range, 2–86). The average follow-up in month was 9 ± 3.87 months (range, 1–14). Six patients (31.60%) were phakic and underwent to combined phacoemulsification associated with pars plana vitrectomy, and thirteen patients (68.42%) were, already, pseudophakic. 100% of the patients in our study had the macular hole closed after a single surgery and maintained it during the follow-up. We obtained primary anatomic success even with the largest macular hole, associated with Alport Syndrome (MLD of 2195 and BD of 2600). The median BCVA in logMAR preoperative was 1.30 ± 0.44 (0.80–2.0), approximately 20/400 in the Snellen chart and the median BCVA in logMAR postoperative was 1.0 ± 0.72 (0.4–3.0) approximately 20/200 and it was statistically significant (p < 0.0001) with median of three lines of improvement (Fig. 1). In five patients vision remained the same, worsened or improved less than three logMAR lines (26.30%), on four patients vision worsened three or more logMAR lines (21.11%) and on ten patients vision improved three or more logMAR lines (52.60%). One patient improved to 0.4 logMAR or 20/50 and it was the best result we achieved in this case report (Fig. 2). In the 19 patients we observed MH MLD ≥ 650 microns preoperatively in 14 patients. The median BCVA in logMAR preoperative was 1.35 and postoperative 1.05, with an improvement of three lines in the logMAR, approximately 20/400 to 20/200 in Snellen chart (P < 0.0001) and was similar with the 19 patients. We had no case of inflammation or infection secondary to the human amniotic membrane.

**Table 1:** Preoperative and postoperative findings in patients with large or refractory macular holes submitted to pars plana vitrectomy and amniotic membrane graft

Patients/age/gender	Diagnosis	Previous surgery	MH BD (µm)	MH MLD (µm)	Time of diagnosis in MO	Status of the lens	BCVA preoperative in logMAR	BCVA PO in logMAR	F/U MO
LPDA/70/F	LMH/PCV	1	2908	915	13	1	1.9	1.6	13
LSMF/81/F	LMH	1	1040	970	3	1	1	1	14
MDS/77/F	LMH	2	930	779	14	1	2	0.6	12
ERF/76/F	LMH	1	1696	616	86	2	1.3	1.6	6
MJSN/62/F	LMH	0	849	733	36	2	1.3	1.3	14
JAA/23/M	LMH/Alport Syndrome	0	2600	2195	48	2	1.3	1	12
IFPP/77/F	LMH	0	2510	1186	24	1	1.3	1	13
TES/85/F	LMH	0	2035	1613	24	1	1.3	1	12
IS/82/F	LMH	0	818	725	72	2	2	1.1	7
AOO/64/M	LMH	0	1219	944	24	1	2	1	6
PL/72/F	LMH	5	1503	1109	60	1	2	1.3	12
ML/56/F	LMH/high Myopia	2	993	988	24	1	1	1	9
CMO/52/F	LMH/high Myopia	1	1116	758	48	2	1.4	1.6	5
DLB/74/F	LMH	1	1528	784	5	2	0.9	2	1
DMFRB/60/F	Reoperation	1	384	333	2	1	0.9	3	10
EDFUC/76/F	Reoperation	1	726	301	13	1	0.8	0.7	7
JBB/61/M	LMH/high Myopia	2	812	409	6	1	2	0.6	6
KBM/46/F	LMH	1	557	403	9	1	1.6	3	9
MFOCN/64/F	LMH	2	489	511	2	1	1.9	0.4	3

*LMH* large macular hole, *PCV* polypoidal choroidal vasculopathy, *MLD* minimum linear diameter, *μm* micron, *logMAR* logarithm of the minimum angle of resolution, *PSP* (1) pseudophakic, *PHACO/PPV* (2) phacoemulsification associated with pars plana vitrectomy, *BCVA* best corrected visual acuity, *F/U* follow-up, *MO*. months

**Fig. 3:**
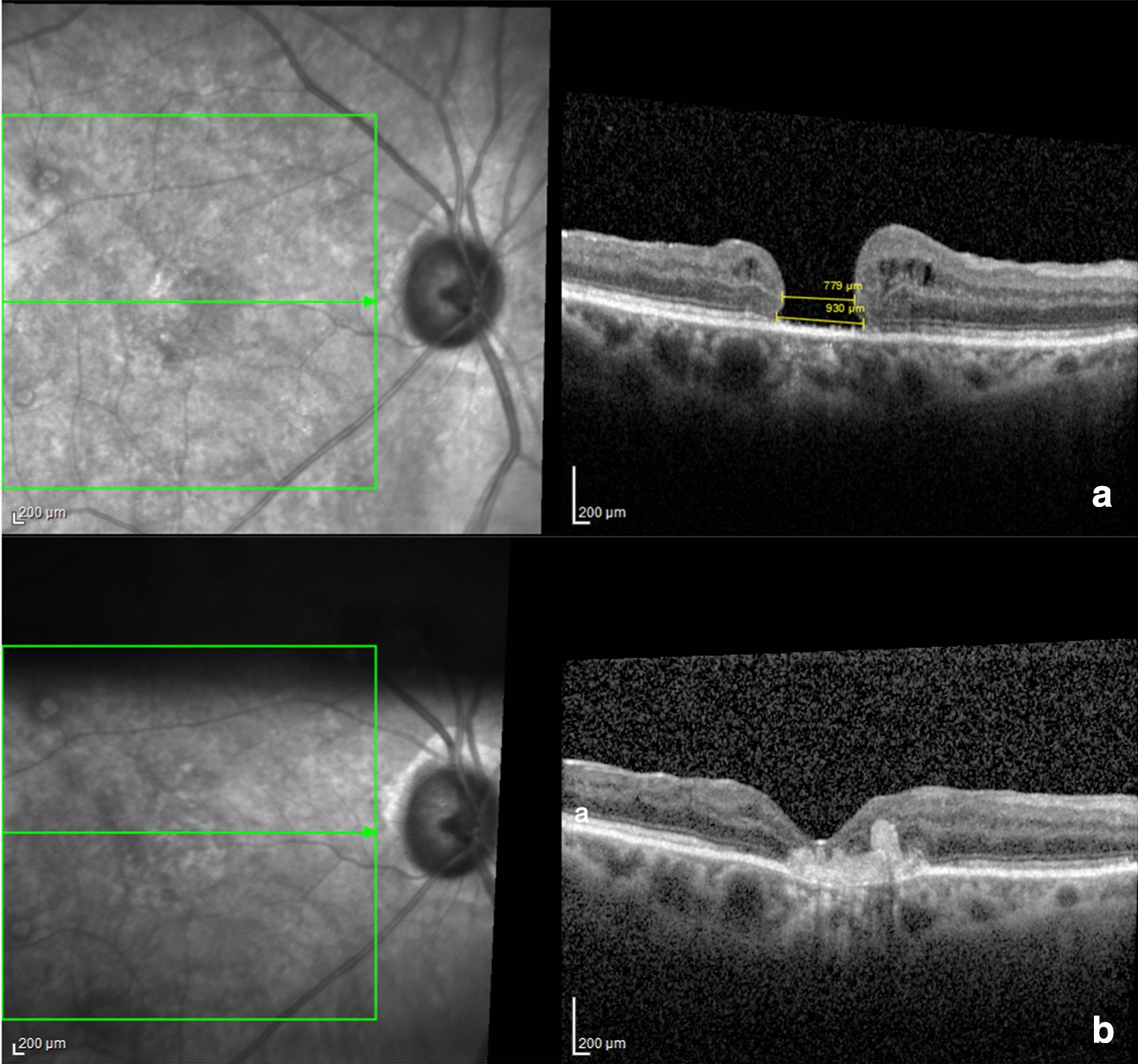
Pre (**a**) and postoperative (**b**) appearance of OCT-SD of a patient submitted to hAM graft, inserted in the macular hole, for the third macular hole reoperation and LMH. **a** Preoperative aspect of the OCT-SD showing MH MLD of 779 microns and MH BD of 930 micron (large macular hole) with BCVA of 1.0 logMAR or approximately 20/200. **b** Postoperative aspect of the OCT-SD showing macular hole closed, formed internal retina with BCVA of 0.6 logMAR or approximately 20/80 in Snellen Chart

In our series, the LMH or recurrent macular holes were resolved in 100% of the patients (19) during the first week after the operation. After the gas endotamponade disappeared, the OCT scan demonstrated the macular hole closure without glial process but with a fully stratified retinal layer over or below the hAM patch (Figs. 2 and 3). During the follow-up period, with the aid of the OCT, it was observed that the neuroretina over or below the hAM plug differentiated to form retinal layers, but this was rarely achieved in the outer layer, such as the external limiting membrane and ellipsoid zone. The mechanism by which hAM promotes the closure of these macular holes is not known, many believe that it serves as a substrate for the inner retina layers as we observed in small and medium MHs and some that can really differentiate into retina layers. This process can probably be associated with the visual acuity improvement found during the follow-up. The healing process was more pronounced in patients where the patch was placed over the macular hole, rather than inserted into the macular hole (Fig. 2), probably due to the fact that there is no manipulation at the edges of the macular hole, preventing damage to the neurosensory retina. In these cases, the hAM seemed to serve as a scaffold for the retina regeneration and the glial aspect of the hAM was observed as a fine layer in the internal retina (Fig. 2). It’s unclear why the use of the hAM at both medium MH MLD subjects worsened the postoperative BCVA.

## Discussion

Human amniotic membrane has been used in superficial ocular pathologies since 1940. Although hAM reduces inflammation and scarring and facilitates epithelialization, there are still uncertainties regarding the integration of the graft and the mechanisms through which it exerts its long-term effects. In vivo transplantation of the hAM to repair a choroidal wound has already been performed in one case, and good integration of the plug to the suprachoroidal structures was observed [20]. The treatment of recurrent macular holes is still a challenge for a retinal surgeon. Caporossi et al. [31] described an anatomical and functional success of the use of hAM at failed macular hole. The failure rate of primary surgery in idiopathic macular holes is less than 10%. It may be due to residual epiretinal traction, insufficient gas tamponade, poor compliance by the patient in keeping prone position, or no obvious cause. All the techniques described at background (repeat fluid gas exchange, endotamponade with silicone oil, radial retinotomy on the MH margins, etc.) have limitations in reoperation or LMH due to initial wide ILM peel, excessive manipulation of the macular hole edges, difficulty in obtaining the harvested ILM tissue for an autologous flap in the periphery, especially in high myopes, dislocation of the ILM flap, impossibility to use the lens capsule in pseudophakic eyes with an open posterior capsule, intraoperative risks of perforation and subretinal hemorrhage, and the long-term risks of compression of the macula from the macular buckle, prolapse to fat, extrusion, and strabismus in macular buckling and scleral imbrication techniques. Some authors have described retinal fibrosis and pigment epithelium dystrophy in the macular area, after internal limiting membrane autologous transplantation for recurrent macular hole, which can affect final visual recovery [31]. The OCT analysis on our patients pre and post-operative did not show these pathological findings, however, it is still unclear in the literature how long it takes for the membrane to disappear and the neural tissue to re-proliferate the hole. On this paper, the hAM grafts remained over the retina during the whole follow-up described at Table 1. In conclusion, all patients had the macular hole closed after the surgery and maintained it during the follow-up. The BCVA remained the same, worsened or improved less than three logMAR lines in five patients (26.30%), on four patients vision worsened three or more logMAR lines (21.11%) and on ten patients vision improved three or more logMAR lines (52.60%). One patient improved to 0.4 logMAR or 20/50 and it was the best result we achieved in this case report (Fig. 2). Our functional results were comparable to the use of autologous retinal transplantation in refractory macular hole [24] where complete anatomic closure of MH by OCT in 36 of 41eyes (87.8%) and VA improved in 52.3% among eyes with anatomic closure were achieved. Autologous retinal transplantation is a more complex technical procedure that involves the removal of the neurosensory retina from a harvest site in the mid-periphery, normally two-disc diameters or larger in size and has more potential for intra and postoperative complications. Human amniotic membrane patches are easy to obtain from the tissue bank, and rather than other substrates used to close macular holes, such as ILM plugs, autologous retinal plugs, or capsular lens fragments, they are also easier to manipulate inside the eye. The limitations of our study are retrospective nature, lack of standardized imaging, individual variations in technique and patient selection and lack of controls. Further prospective and control group studies are necessary to determine the efficacy of this new technique, and more intraoperative and postoperative analyses would be useful to understand the interaction between the amniotic membrane and the retina. Inducing retinal ingrowth and stimulating regeneration of the retinal inner layers, the external limiting membrane, and the ellipsoid zone in these patients is a new frontier for vitreoretinal surgery.

## Data Availability

The datasets used and/or analysed during the current study are available from the corresponding author on reasonable request.
